# 

**Published:** 1888-12

**Authors:** 


CONTENTS.

VOL. 35 No. 12.

| | PAGE. |
|---|---|
| Last of the Series of 1888 | 265 |
| Mind Cure Matter, etc | 266 |
| Magnetic Hygiene | 269 |
| What to Eat | 271 |
| The Onion | 274 |
| Good Living and the Brain | 274 |
| Plantains and Bananas | 275 |
| Salt as a Medicine | 277 |
| Anecdote of H. Ward Beecher | 278 |
| A Good Work | 279 |
| Cuba's Two Meals a Day | 279 |
| Psycometric Power | 280 |
| Calisthenic Exercises for Girls | 281 |
| A Soldier Somnambulist | 281 |
| Treatment of Ingrowing Nail | 282 |
| Infant Feeding | 282 |
| Over-Eating | 283 |
| Book Notices | 284 |
| Maggie's Big Toe | 285 |
| Household | 286 |
| Miscellaneous | 287 |

INDEX TO ADVERTISEMENTS OF HALF A PAGE AND OVER.

| | PAGE. |
|---|---|
| Murdock's Liquid Food Co | I |
| Stevensville Mills | II |
| The Herb Cure Co | II |
| The Farm and Household Cyclopædia | III |
| Hollow Suppositories | IV |
| Lactopeptine | V |
| The Family Cyclopædia of Useful Knowledge | VI |
| The Banner of Life and Home Physician | VIII |
| Rio Chemical Co | X |
| Davis' Elixir of Health | XI |

